# Time course of endothelial dysfunction markers and mortality in COVID‐19 patients: A pilot study

**DOI:** 10.1002/ctm2.283

**Published:** 2021-03-01

**Authors:** Francesco Vieceli Dalla Sega, Francesca Fortini, Savino Spadaro, Luca Ronzoni, Ottavio Zucchetti, Marco Manfrini, Elisa Mikus, Alberto Fogagnolo, Francesca Torsani, Rita Pavasini, Luisa Marracino, Marco Verri, Luca Morandi, Emanuele D'Aniello, Carlo Alberto Volta, Gianluca Campo, Roberto Ferrari, Paola Rizzo, Marco Contoli

**Affiliations:** ^1^ Maria Cecilia Hospital GVM Care & Research, Cotignola Ravenna Italy; ^2^ Intensive Care Unit, Department of Morphology Surgery, and Experimental Medicine University of Ferrara Ferrara Italy; ^3^ Respiratory Section, Department of Morphology Surgery and Experimental Medicine University of Ferrara Ferrara Italy; ^4^ Cardiovascular Institute Azienda Ospedaliero‐Universitaria di Ferrara, Cona Ferrara Italy; ^5^ Department of Morphology Surgery and Experimental Medicine University of Ferrara Ferrara Italy; ^6^ Laboratory for Technologies of Advanced Therapies University of Ferrara Ferrara Italy

To the Editor:

Previous studies suggested that the endothelial dysfunction is associated with the severity and mortality of patients affected by coronavirus disease 2019 (COVID‐19). However, the temporal evolution of markers of endothelial dysfunction during the progression of the disease and the relative relationship with the exitus are less established.^1^, ^2^

The present study is a subanalysis from the “Pro‐thrombotic status in patients with SARS‐CoV‐2 infection” study (ATTAC‐Co, https://www.clinicaltrials.gov/ identifier NCT04343053) focused on biomarkers of endothelial dysfunction. The ATTAC‐Co is a single‐center, prospective study including 54 patients admitted to hospital for moderate‐severe respiratory failure and positive for severe acute respiratory syndrome coronavirus 2 (SARS‐CoV‐2) infection. SARS‐CoV‐2 infection was confirmed by reverse transcriptase‐polymerase chain reaction assay from nasopharyngeal swab specimen. In COVID‐19 patients, symptoms appeared 9 ± 2 days before admission. The control group included 11 patients admitted to Pulmonology Unit with respiratory failure and negative to SARS‐CoV‐2 infection. Endothelial dysfunction biomarkers (endothelin‐1, endoglin, sE‐selectin, thrombomodulin, soluble vascular cell adhesion molecule 1 [sVCAM‐1], and von Willebrand factor [vWF]) were measured in three different blood samples in COVID‐19 patients (inclusion [T1], after 7 ± 2 days [T2], and 14 ± 2 days [T3]). These patients received 4000‐8000 IU enoxaparin twice per day. On the contrary, one single blood sample withdrawal was performed at inclusion in controls. The aims of the present analysis were the comparison between cases versus controls, and between survivors versus nonsurvivors. The protocol was approved by the corresponding Ethics Committee, and all patients provided written informed consent. Details on the clinical trial, assays, and statistics are provided in the Supplementary Material.

Table 1 reports clinical, laboratory, and biomarker data of control and COVID‐19 patients at entry. Baseline characteristics did not differ between COVID‐19 patients and controls (Table 1). Table 2 summarizes the molecular function of endothelial dysfunction biomarkers. COVID‐19 patients showed higher values of C‐reactive protein, high‐sensitivity troponin, IL‐6, and TNF‐α. Regarding endothelial biomarkers, sVCAM‐1 and thrombomodulin were significantly higher (Table 1 and Figure 1A).

**TABLE 1:** Baseline characteristics

	Controls (n = 11)	Cases (n = 54)	*P*1	Survivors (n = 38)	Nonsurvivors (n = 16)	*P*2
Age, years	70 [66‐80]	65 [57‐73]	.244	62.5 [55‐71]	72.5 [65‐78]	**.003**
Male sex, no. (%)	8 (73)	40 (74)	.999	28 (74)	12 (75)	.602
BMI, kg/m^2^	24.3 [21.7‐27.0]	26.4 [24.2‐30.2]	.169	26 [24‐29.4]	28.5 [26.1‐31]	.218
**Comorbidities, no. (%)**						
Hypertension	5 (45)	30 (55)	.741	20 (53)	4 (25)	.078
Dyslipidemia	5 (45)	11 (20)	.120	7 (18)	4 (25)	.713
Former smoker	4 (36)	16 (30)	.725	9 (24)	7 (44)	.194
Diabetes	3 (27)	7 (13)	.353	5 (13)	2 (13)	.999
Prior MI	0 (0)	3 (6)	.999	1 (3)	2 (12)	.206
Prior coronary revascularization	0 (0)	3 (6)	.999	1 (3)	2 (12)	.206
Prior CVA	1 (9)	2 (4)	.432	2 (5)	0 (0)	.491
Peripheral artery disease	2 (18)	8 (15)	.673	3 (8)	5 (31)	**.041**
Chronic kidney disease	4 (36)	13 (24)	.400	5 (13)	8 (50)	**.004**
**Home medical therapy, no. (%)**						
Aspirin	1 (9)	5 (9)	.999	2 (5)	3 (19)	.147
ACE inhibitors	4 (36)	21 (39)	.999	13 (34)	8 (50)	.362
Beta‐blockers	2 (18)	12 (22)	.999	6 (16)	6 (37)	.148
Calcium channel blockers	1 (9)	9 (17)	.999	4 (10)	5 (31)	.105
Statins	2 (18)	9 (17)	.999	4 (10)	5 (31)	.105
**Respiratory parameters at inclusion**						
P/F ratio	140 [80‐220]	131 [79‐210]	.354	161 [90‐229]	107 [76‐131]	.097
PaO2, mmHg	87 [60‐120]	85 [65‐119]	.423	90 [68‐126]	75 [65‐101]	.287
PaCO2, mmHg	42 [25‐55]	41 [26‐52]	.357	39 [35‐51]	48 [35‐62]	.165
pH	7.4 [7.4‐7.5]	7.5 [7.4‐7.5]	.854	7.5 [7.4‐7.5]	7.4 [7.3‐7.5]	.083
Laboratory data at inclusion						
WBC (u × 10^3^/L)	12.1 [9.6‐15.1]	9.1 [5‐15]	.121	9.1 [6.5‐12.7]	9.1 [7.3‐11.8]	.788
Lymphocytes (u × 10^3^/L)	0.86 [0.71‐1.75]	0.80 [0.52‐1.06]	.217	0.83 [0.52‐1.11]	0.72 [0.56‐0.90]	.404
Hemoglobin, g/dL	12.1 [11.4‐13.7]	10.7 [9.6‐12.3]	.071	11 [9.6‐12.3]	10.5 [9.7‐12.1]	.968
Creatinine clearance, mg/dL	87.2 [45.6‐108.6]	88.1 [74.1‐106.2]	.391	96 [80.5‐109.5]	75.8 [60‐91.2]	**.024**
Platelets (u × 10^3^/L)	253.5 [162‐277]	293 [245‐382]	.072	311 [264‐396]	247 [174‐326]	**.036**
Fibrinogen, mg/dL	464 [452‐802]	708 [576‐823]	.275	705 [576‐823]	765 [584‐842]	.356
D‐dimer, mg/L FEU	0.85 [0.70‐1.7]	2.3 [1.2‐4.2]	.064	1.85 [1.15‐3.95]	2.8 [1.8‐4.3]	.173
C‐reactive protein, mg/dL	5.7 [3.4‐10.3]	12.2 [5.4‐20.5]	.087	9.9 [2.7‐15.6]	16.6 [12.5‐29.1]	**.004**
HS troponin I, ng/L	9 [3‐18]	15.5 [8‐52]	.001	9.5 [8‐24]	34 [15.5‐96.5]	**.002**
IL‐6, pg/mL	7.8 [1.1, 32.7]	37.7 [9.7, 146.0]	.014	35.0 [7.1, 148.5]	41.5 [22.9, 127.8]	.543
TNF‐α, pg/mL	11.3 [7.9, 33.6]	32.5 [21.8, 54.6]	.041	31.82 [21.5, 53.8]	42.6 [27.4, 58.8]	.343
**Biomarkers at inclusion**						
Soluble endoglin, pg/mL	1045.2 [696.6, 1443.3]	1131.9 [523.3, 1488.0]	.944	921.3 [432.6, 1435.3]	1408.9 [884.6, 1902.0]	**.023**
Endothelin‐1, pg/mL	5.8 [5.8, 14.9]	7.2 [2.8, 9.8]	.384	5.8 [2.8, 8.5]	8.5 [5.4, 11.1]	.143
sE‐selectin, ng/mL	17.8 [16.6, 38.2]	23.6 [18.9, 35.8]	.441	23.0 [18.0, 39.4]	24.0 [22.4, 30.4]	.437
sVCAM‐1, ng/mL	750.8 [643.2, 966.1]	1087.9 [835.4, 1614.4]	.010	986.38 [789.6, 1392.7]	1574.3 [1331.0, 1947.7]	**.003**
Thrombomodulin, ng/mL	5.8 [4.5, 7.3]	8.6 [5.7, 13.9]	.031	8.3 [5.5, 11.6]	12.6 [7.1, 17.6]	.153
vWF, μg/mL	41.3 [17.9, 57.7]	43.0 [27.1, 84.6]	.146	44.5 [27.4, 78.4]	38.8 [26.8, 86.0]	.999

*Note*. Data are reported as median [interquartile range]. *P*1: for the comparison between controls versus cases. *P*2: for the comparison between survivors versus nonsurvivors.

Abbreviations: ACE, angiotensin converting enzyme; BMI, body mass index; COPD, chronic obstructive disease; CVA, cerebrovascular accident; FEU, fibrinogen equivalent unit; HS, high sensitivity; MI, myocardial infarction; WBC, white blood cells.

**TABLE 2:** Biological characteristics of the endothelial dysfunction biomarkers

Biomarker	Molecular function	Endothelial dysfunction
Soluble endoglin	Endoglin is a coreceptor of the transforming growth factor β receptor. It regulates vascular remodeling, angiogenesis, and the activity of endothelial nitric oxide synthase	Soluble form of endoglin is released after shedding, and it is a marker of endothelial injury, activation, and inflammation
Endothelin‐1	Produced by vascular endothelial cells as a precursor is cleaved to be secreted in its active form. Endothelin‐1 binds to its receptors exerting a potent vasoconstrictor	High levels of circulating endothelin‐1 are associated with the impairment of vascular tone regulation
sE‐selectin	Expressed on the membrane of activated endothelial cells, and promotes adhesion of leukocytes	Circulating E‐selectin reflects endothelial activation or damage
sVCAM‐1	Expressed on the membrane of activated endothelial cells, and promotes adhesion of leukocytes	Circulating sVCAM‐1 reflects endothelial activation or damage
Soluble thrombomodulin	Expressed on the membrane endothelial cells, plays a role as a protein C cofactor and possess anticoagulant activity	Released from injured endothelial cells is a marker of endothelial cell damage
von Willebrand factor (vWF)	Expressed in the endothelial cells, and mediates platelets adhesion to the endothelium	vWF is released when endothelial cells are damaged and reflects a prothrombotic status

**FIGURE 1:**
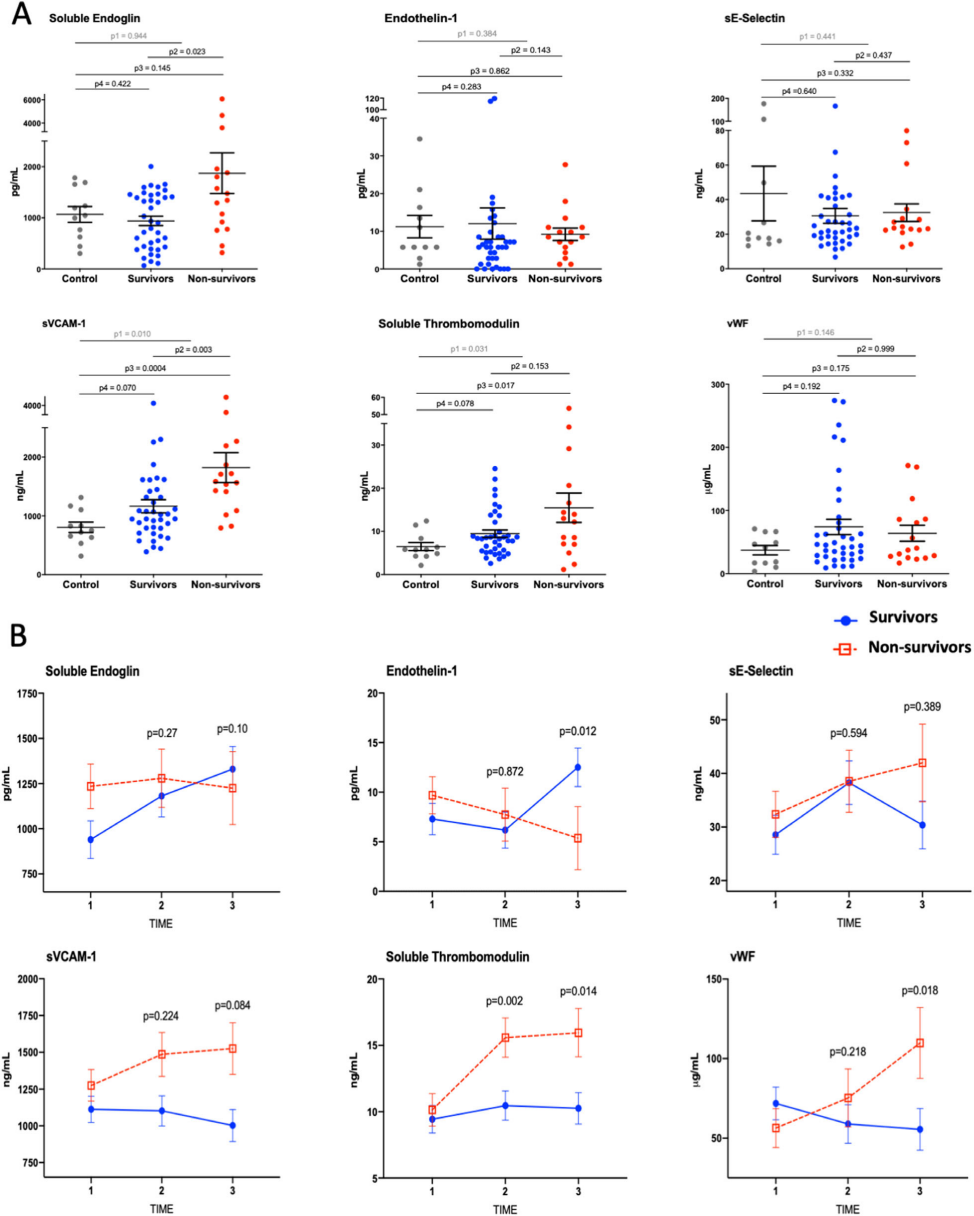
A, Comparisons of endothelial dysfunction markers at baseline in controls without SARS‐CoV‐2 and in survivor versus nonsurvivor COVID‐19 patients. Points indicate individual values and horizontal bars show means. The whiskers are the SEMs. *P*1: For the comparison between controls versus all cases; *P*2: for the comparison between survivors versus nonsurvivors; *P*3: for the comparison between controls versus survivors; *P*4: for the comparison between controls versus nonsurvivors. B, Temporal changes in endothelial dysfunction markers in patients with COVID‐19, survivors versus nonsurvivors. The graphs represent the marginal effects stratified by outcome. The squares and the circles represent the marginal effect at the specific time point. The whiskers are the SEMs. The lines between squares and circles represent the trend over time. The *P*‐values represent the difference of marginal effects in the single time point between groups. Blue lines and circles: patients who survived. Red dashed lines and squares: patients who did not survive. N at each time point were: T1 = 54; T2 = 48; T3 = 43

In the follow‐up, 16 out of 54 (30%) COVID‐19 patients died. As expected, several baseline characteristics and laboratory findings differed between nonsurvivors and survivors (Table 1). As reported by others, there was a higher frequency of CKD and higher levels of C‐reactive protein,^3^ but not higher number of current smokers in nonsurvivors compared to survivors. Different from other published studies, in our set of data PAD was more frequent among nonsurvivors. Endoglin and sVCAM‐1 were significantly higher at inclusion in nonsurvivors versus survivors (Table 1 and Figure 1A). Interestingly, sVCAM‐1 in control versus nonsurvivors remains strongly significant, while it loses significance when only the survivors’ group is compared to controls (Figure 1A). A similar trend is also observed for thrombomodulin (Figure 1A). This underlines the increase of these two biomarkers in the nonsurvivors’ group.

Figure 1B illustrates the time course of the biomarkers in survivors versus nonsurvivors corrected for potential confounding variables. In nonsurvivors, thrombomodulin and vWF significantly increased over time (Figure 1B). sVCAM‐1 displayed a similar trend but without reaching statistical significance (*P* = .08) (Figure 1B). Consistent with other studies, we found high levels of vWF both in COVID‐19 and controls compared to normal values.^4^, ^5^, ^6^ Furthermore, vWF levels increased over time in nonsurvivors versus survivors, in agreement with other reports of high mortality and worse prognosis in patients with higher levels of vWF.^5^, ^7^ In contrast, endothelin‐1 remained stable in nonsurvivors but increased over time in survivors (Figure 1B). The remaining biomarkers of endothelial dysfunction did not show significant variations between survivors and nonsurvivors over their time courses (Figure 1B and Table S1). Univariate Cox regression analyses showed that endoglin, thrombomodulin, and sVCAM‐1 levels were associated with death (Table S2). Multivariate analysis, including the variables associated with death on univariate analysis listed in Table S2, revealed that only sVCAM‐1 was independently associated with mortality (Table S2). Figure 2A shows that, on admission, patients with levels of sVCAM‐1 higher than median (>1088 ng/mL) versus those with levels lower than median (<1088 ng/mL) have higher mortality rate. The predictive mortality value of sVCAM‐1 is confirmed by the ROC analysis yielding in a high area under the curve (AUC = 0.823) (Figure 2B). This is true also for patients younger than 75 years (Figure S1B,C).

**FIGURE 2:**
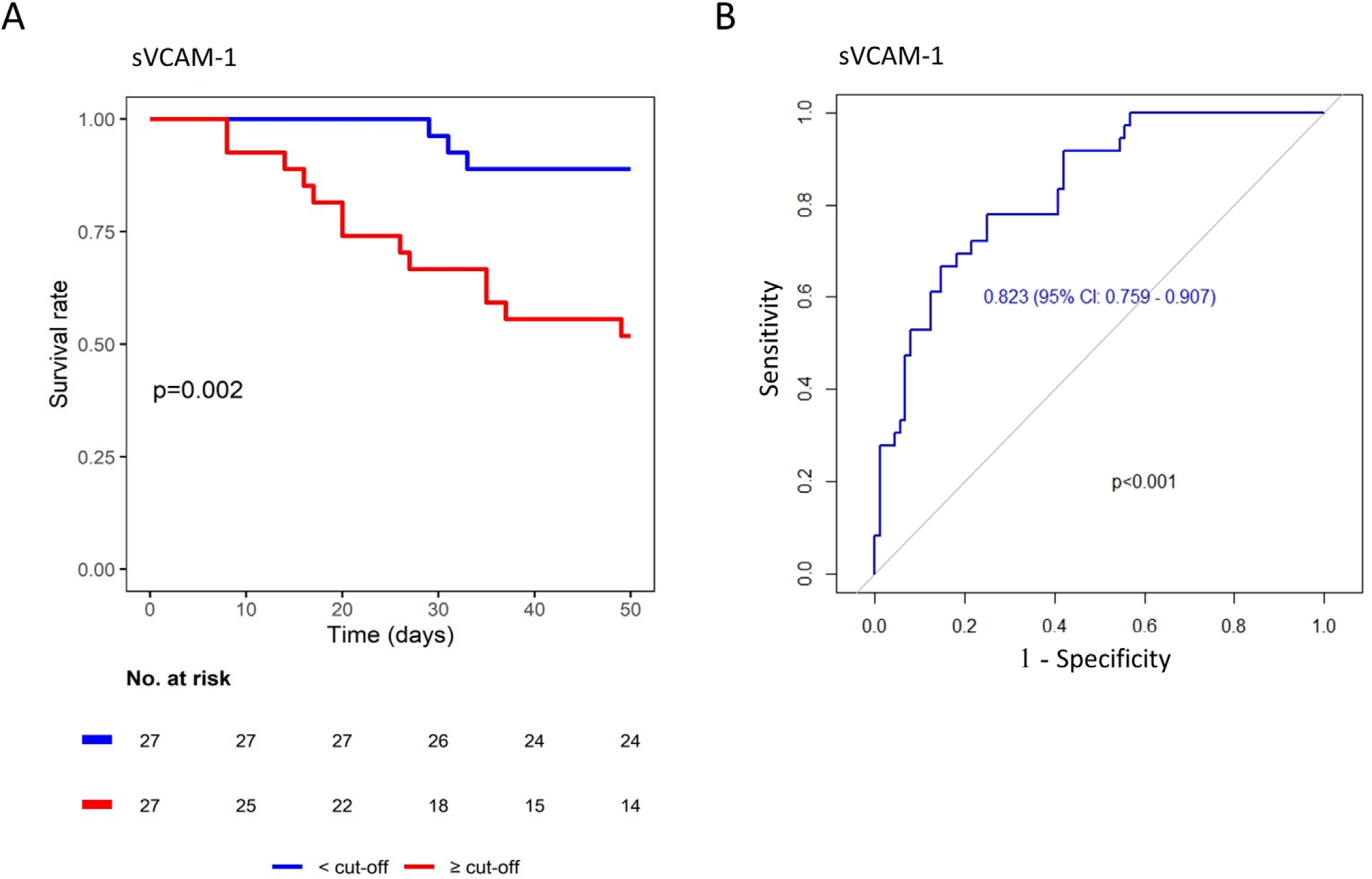
A, Kaplan‐Meier analysis. Patients were stratified by soluble vascular cell adhesion molecule 1 (sVCAM‐1) median concentration in blood at hospital admission. Plot shows *P*‐value of the log‐rank test between groups. B, Time‐dependent ROC analysis of sVCAM‐1 blood concentration toward patients outcomes, showing area under the curve (AUC) with 95% confidence interval and its associated *P*‐value

Our data confirm and expand previous evidence on the role of endothelial dysfunction in COVID‐19 patients. First, COVID‐19 patients have higher levels of thrombomodulin and sVCAM‐1 compared to controls with same clinical presentation but without SARS‐CoV‐2 infection. This supports the concept that COVID‐19 pneumonia specifically affects the endothelium.^2^ Second, endoglin, an adhesion molecule expressed by endothelial cells in response to inflammation, never investigated before in the context of COVID‐19, is associated with death. Although multivariable analysis did not confirm its independent prognostic role, we believe that this finding further supports how the link between inflammatory storm and endothelial dysfunction should be considered crucial in COVID‐19 pathogenesis. Third, sVCAM‐1 is the only biomarker that, at multivariate analysis, remained independently associated to mortality. Older age is associated to increased cardiovascular comorbidities and is a risk factor for COVID‐19 mortality.^8^ However, sVCAM‐1 predictive value is highlighted by survival and ROC analyses, and is maintained in younger patients (<75 years). The early increase of VCAM‐1 may promote leukocytes recruitment, thus determining increased vascular permeability, tissue damage, and the release of further proinflammatory cytokines, all eventually contributing to mortality. These data have clinical implications. Dexamethasone in vitro contrasts the expression of sVCAM‐1 in endothelial cells,^9^ and the RECOVER trial showed that dexamethasone treatment reduces mortality in hospitalized COVID‐19 patients.^10^ It is plausible that dexamethasone reduces mortality, at least in part, also by contrasting sVCAM‐1‐induced endothelial activation and leukocyte recruitment. Fourth, surprisingly, endothelin‐1 increased over time in COVID‐19 survivors. This finding is counterintuitive as, under normal conditions, endothelin‐1 participates in the regulation of vascular tone contrasting the effect of nitric oxide. Endothelin‐1 is elevated in pulmonary arterial hypertension, a condition that is treated by endothelin‐1 receptor antagonists.^11^ Elevated pulmonary arterial hypertension has been observed in COVID‐19 patients and the constant increase found in the survivors may be linked to this.^12^ On the other hand, endothelin‐1 is also linked to angiogenesis,^13^ which has been observed in the lung of COVID‐19 patients.^14^ It is tempting to speculate that higher endothelin‐1 levels promote angiogenesis in the survivors even though the clinical significance of angiogenesis in the lung of COVID‐19 patients remains to be established.

Our report is just a proof‐of‐concept, hypothesis‐generating effort that has several limitations. The results are not applicable to asymptomatic or mild‐symptomatic COVID‐19 patients. Although we assessed three different time points, we cannot describe the alterations in endothelial function between viral infection and the admission to the hospital. In addition, it should be noted that the sample size of our pilot study is small and further investigations in larger population are clearly on demand. Furthermore, this report focuses on endothelial dysfunction, thus the role of other cardiovascular parameters has not been discussed. In conclusion, we performed an analysis of a broad panel of biomarkers of endothelial dysfunction at different time points in COVID‐19 patients. We observed important changes over time of the biomarkers, and in particular those of sVCAM‐1 seem to be strongly related to mortality.

## CONFLICT OF INTEREST

The authors declare that there is no conflict of interest.

## DATA AVAILABILITY STATEMENT

The data that support the findings of this study are available from the corresponding author upon reasonable request.

## Supporting information

Supporting Information
