# Supplementary material for: Time course of endothelial dysfunction markers and mortality in COVID‐19 patients: A pilot study
Source: Clin Transl Med. 2021 Mar 1;11(3):e283. doi: 10.1002/ctm2.283 (PMC7919132; doi:10.1002/ctm2.283)
Supplement: Supplementary file 1 — Supporting Information [file CTM2-11-e283-s001.docx]

**The course of endothelial dysfunction markers and mortality in COVID-19 patients: a pilot study**

Supplemental Online

**Methods**

***Study design***

“Pro-thrombotic status in patients with SARS-CoV-2 infection” (ATTAC-Co) study is an investigator-initiated, prospective cohort, single-center study recruiting consecutive patients admitted to hospital because of respiratory failure due to COVID-19 between April and May 2020. The patients were recruited at the Respiratory and Intensive Care Units dedicated to the management of COVID-19 patients (COVID-19 Units) of the “Azienda Ospedaliera Universitaria di Ferrara” (Cona [FE], Italy) while the analyses were performed at the Translational Research Center of Maria Cecilia Hospital (Cotignola, Italy). An electronic case report form (CRF) was used to collect demographic characteristics, previous medical history, comorbid conditions, and concomitant treatments. At 3 different time points (inclusion [T1], after 7±2 days [T2] and after 14±2 days [T3]) the following clinical data were recorded: symptoms, physical examination, vital parameters (including arterial blood gas test) laboratory and imaging data (according to clinical indications), needs of oxygen supplementation, and of non-invasive or invasive mechanical ventilations and relative setting parameters (obtained either at respiratory unit or intensive care unit), pharmacological treatments and outcomes. In-hospital occurrence of adverse events was daily monitored, whereas after hospital discharge follow-up visits were performed every 30 days (last update June 30^th^, 2020). At T1, T2 and T3 of the study, blood samplings for the evaluation of several biomarkers of platelet function, coagulation cascade activation, fibrinolysis, endothelial dysfunction, and inflammation were withdrawn. Here we report the main findings related to endothelial dysfunction. The protocol was approved by the corresponding Ethics Committee (Comitato Etico di Area Vasta Emilia Centro, Bologna, Italy). All patients provided written informed consent. In case of unconsciousness, the informed consent was signed by the next of kin or legal authorized representative. The study is registered at www.clinicaltrials.gov with the identifier NCT04343053.

***Study population***

A total of 65 patients participated to the study. Inclusion criteria were: i) age >18 years; ii) confirmed SARS-CoV-2 infection; iii) hospitalization for moderate-severe respiratory failure; iv) need for mechanical ventilation or oxygen support. Moderate-severe respiratory failure was defined as a PaO2/FiO2 (P/F) ratio ≤ 200 mmHg at first medical contact. This resulted in n=54 patients included in the study. Patients admitted to hospital between April and May 2020 with similar clinical characteristics in term of respiratory failure presentation, but negative for SARS-CoV-2 infection were included as controls (n = 11). The causative micro-organisms of the respiratory failure of non-COVID-19 patients are shown in Table S4. Cases and controls were excluded from the study if any of the following criteria applied: prior administration of P2Y12 inhibitors, or anticoagulant drugs, known disorder of coagulation or platelet function and/or chronic inflammatory disease. SARS-CoV-2 infection was confirmed by reverse-transcriptase-polymerase-chain-reaction assay (Liaison MDX, Diasorin, Saluggia, Italy) from nasopharyngeal swab specimen. Clinical management was in accordance with current guidelines and specific recommendations for COVID-19 pandemic by Health Authorities and Scientific Societies.

***Blood samples***

Blood samplings were performed from an antecubital vein using a 21-gauge needle or from central venous line. Blood was collected in the early morning at least 12 hours after last administration of anticoagulant drugs. The first 2 to 4 mL of blood were discarded, and the remaining blood was collected in EDTA tubes. Tubes were centrifuged, plasma were collected, aliquoted and immediately stored at -80 °C.

***Quantification of plasma marker of endothelial dysfunction***

Based on previous studies (Leite *et al.*, 2020), the following circulating biomarkers were selected as surrogates of endothelial dysfunction: endothelin-1, endoglin, sE-selectin, thrombomodulin, sVCAM-1, and von Willebrand Factor. The plasma levels of endothelin-1, endoglin, E-selectin, thrombomodulin, sVCAM-1, were determined with the bead-based multiplex immunoassay Milliplex Map (EMD Millipore Burlington, MA, USA). Due to the death or transfer of some patients it was not possible to collect blood samples at each time point. As a result, n at each time point were: T1 = 54; T2 = 48; T3 = 43. Samples were processed following manufacturer's instructions, data were analyzed by MAGPIX system provided with the xPONENT Software (Luminex, Thermo Fisher Scientific, Waltham, MA, USA). Plasma levels of von Willebrand Factor (vWF) were quantified by ELISA (Thermo Fisher Scientific, Waltham, MA, USA). Coefficient Variations (CVs) % of the quantification for each biomarker, as defined by the manufacturers, are shown in Table S3.

***Statistical analysis***

Continuous variables are presented as mean [standard deviation] or median [interquartile range] and categorical variables as counts and proportions (%). Normality assumption of sampled data was checked both visually by inspecting the QQ plots and through Shapiro-Wilk normality test. For continuous variables, the differences were compared between groups using the one-way analysis of variance and the Kruskal Wallis test for parametric and non-parametric data, respectively.

To model continuous response variable represented by serial measurements over time within subject we set up a generalized least squares model taking into account correlations between measurements on the same subject to have optimal model fits and honest inference. Correlation type was selected by fitting the same model with different correlation structure and selecting the model with lower Akaike's information criterion (AIC). For each response variable a full model for overall population and stratified by survival status containing potential confounders was built. Backward stepwise selection was carried out to remove poor informative variable evaluating AIC and log-likelihood ratio test significance. Once models were optimized, estimated marginal effect were calculated and plotted as response variable predicted values against time.

Cox proportional hazards regression modelling was used to analyze the effect of several variables on death (all-cause mortality). All baseline variables were tested in univariate model, and those found to be significative (p<0.05) were included in adjusted multivariate Cox regression analysis. Results are reported as hazard ratios with associated 95% confidence intervals (CIs). The multicollinearity was examined using the variance inflation factor (VIF) and variables with VIF > 5 were excluded by the same multivariable model. Variable selection was performed by a backward stepwise algorithm based on Akaike’s information criterion minimization. Cross-validation was performed to validate the models obtained. The p-value related to the likelihood ratio was calculated, along with Harrel’s C-index. Empirical cut off points were determined for the outcome of interest applying the change-point method to the survival models. To evaluate the performance of the biomarkers selected model discrimination capability was assessed using time dependent AUC (AUC-tdROC).

The analysis was performed by MM with R version 3.5.1 (R Foundation for Statistical Computing, Vienna, Austria) and STATA.

**Table S1. Endothelial function biomarkers at different time points in survivors and non-survivors**

|  |  | **T1** | **p1** | **T2** | **p2** | **T3** | **p3** |
| --- | --- | --- | --- | --- | --- | --- | --- |
| **Endoglin**  **(pg/mL)** | survivors | 921.28 [432.57, 1435.34] | **0.023** | 1147.02 [512.47, 1554.32] | 0.551 | 1233.48 [646.54, 1748.51] | 0.786 |
|  | non-survivors | 1408.90 [884.59, 1902.05] |  | 1210.08 [929.59, 1533.75] |  | 1129.88 [626.98, 1818.92] |  |
| **Endothelin-1**  **(pg/mL)** | survivors | 5.77 [2.82, 8.51] | 0.143 | 3.58 [2.04, 9.16] | 0.669 | 5.77 [2.82, 11.98] | 0.838 |
|  | non-survivors | 8.51 [5.41, 11.06] |  | 7.47 [1.04, 10.27] |  | 8.51 [2.43, 8.83] |  |
| **sE-Selectin**  **(ng/mL)** | survivors | 23.05 [18.02, 39.42] | 0.437 | 25.44 [17.78, 58.80] | 0.927 | 24.23 [16.09, 33.13] | 0.288 |
|  | non-survivors | 23.98 [22.45, 30.37] |  | 27.36 [20.11, 36.48] |  | 27.12 [23.72, 35.81] |  |
| **sVCAM-1**  **(ng/mL)** | survivors | 986.38 [789.56, 1392.67] | **0.003** | 890.34 [742.12, 1144.82] | **0.026** | 956.50 [772.76, 1333.70] | **0.008** |
|  | non-survivors | 1574.35 [1331.02, 1947.67] |  | 1535.57 [1032.60, 2058.10] |  | 1497.86 [1210.38, 2225.84] |  |
| **Thrombomodulin**  **(ng/mL)** | survivors | 8.31 [5.50, 11.56] | 0.153 | 7.58 [6.56, 11.38] | **0.005** | 7.88 [6.62, 11.49] | **0.024** |
|  | non-survivors | 12.58 [7.07, 17.60] |  | 16.62 [10.99, 20.74] |  | 11.50 [10.25, 17.78] |  |
| **vWF**  **(μg/mL)** | survivors | 44.53 [27.41, 78.39] | 0.999 | 43.55 [20.35, 77.34] | 0.094 | 29.67 [12.33, 49.93] | 0.055 |
|  | non-survivors | 38.82 [26.84, 85.99] |  | 64.68 [49.79, 77.99] |  | 87.25 [36.76, 172.29] |  |

p: comparison between survivors vs non-survivors at the three different time-point

**Table S2. Association between biomarkers and mortality**

|  | **Univariable** | |  | **Multivariable** | |  |
| --- | --- | --- | --- | --- | --- | --- |
|  | **HR** | **95% CI** | **p** | **HR** | **95% CI** | **p** |
| Age | 2.58 | 1.19 – 5.59 | **0.016** | 6.96 | 1.65 – 29.24 | **0.0081** |
| Former smoker | 3.00 | 1.10 – 8.24 | **0.032** | 6.35 | 1.27 – 31.63 | **0.024** |
|  |  |  |  |  |  |  |
| **Comorbidities** |  |  |  |  |  |  |
| Peripheral artery disease | 3.53 | 1.21 – 10.27 | **0.021** |  |  | 0.111 |
| Chronic kidney Disease | 4.33 | 1.62 – 11.58 | **0.0035** | 3.75 | 1.08 - 12.99 | **0.037** |
|  |  |  |  |  |  |  |
| **Laboratory** |  |  |  |  |  |  |
| Platelets (u x10^3^/L) | 0.49 | 0.26 – 0.92 | **0.026** |  |  | 0.427 |
| C-reactive protein (mg/dl) | 2.22 | 1.506 – 3.27 | **0.0001** | 2.97 | 1.285 – 6.87 | **0.011** |
|  |  |  |  |  |  |  |
| **Biomarkers** |  |  |  |  |  |  |
| Endoglin | 2.75 | 1.17 – 6.50 | **0.021** |  |  | 0.492 |
| sVCAM-1 | 2.28 | 1.19 – 4.39 | **0.013** | 2.79 | 1.35 – 5.78 | **0.0056** |
| Thrombomodulin | 2.18 | 1.17 – 4.04 | **0.014** |  |  | 0.310 |
|  |  |  |  |  |  |  |

HR: Hazard Ratio; CI: Confidence Interval

**Table S3. Intra- and Inter-assay %CV for each biomarker.**

| **Biomarker** | **Intra-assay %CV** | **Inter-assay %CV** |
| --- | --- | --- |
| sVCAM1 | <15% | <20% |
| sE-selectin | <10% | <20% |
| Soluble Thrombomodulin | <10% | <20% |
| sEndoglin | <10% | <20% |
| Endothelin-1 | <10% | <20% |
| vWF | <10% | <12% |

**Table S4. The causative micro-organisms of the respiratory failure of non-COVID-19 patients**

|  | **Controls**  **(n=11)** |
| --- | --- |
| **Causative micro-organism, no. (%)** |  |
| Gram-positive | 2 (18) |
| Gram-negative | 4 (37) |
| Mixed Gram-positive and negative | 1 (9) |
| Other | 1 (9) |
| Unknown | 3 (27) |

**
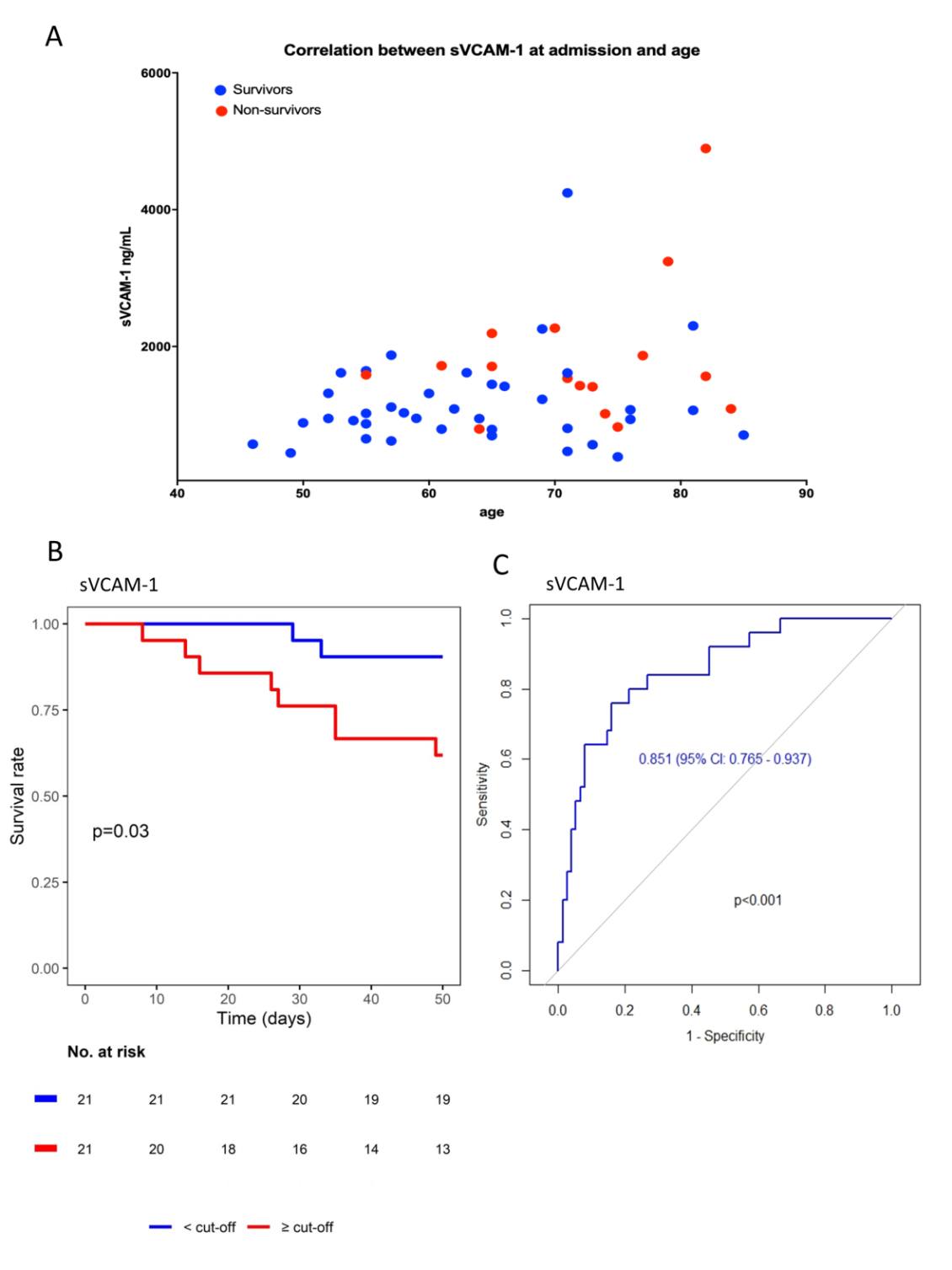
**

**Figure S1 –** (A) Correlation between sVCAM-1 and age. Red circles indicate individuals who did not survive, blue circles indicate individuals who survived. Correlation was evaluated with Pearson coefficient: r = 0.211; p = 0.124. (B) Kaplan–Meier analysis. Patients < 75 age (n = 42) were stratified by sVCAM-1 median concentration in blood at hospital admission. Plot shows p value of the log rank test between groups. (C) Time dependent ROC analysis of sVCAM-1 blood concentration toward patients (<75 age) outcomes, showing AUC with 95% confidence interval and its associated p value.
